# Riociguat prevents fibrotic tissue remodelling and improves survival in salt-sensitive Dahl rats

**DOI:** 10.1186/1471-2210-11-S1-P28

**Published:** 2011-08-01

**Authors:** Sandra Geschka, Axel Kretschmer, Yuliya Sharkovska, Oleg V Evgenov, Andreas Hucke, Bettina Lawrenz, Berthold Hocher, Johannes-Peter Stasch

**Affiliations:** 1 Cardiology Research, Bayer HealthCare, Wuppertal, Germany; 2 Global Biomarker, Bayer HealthCare, Wuppertal, Germany; 3 Institute of Nutritional Science, University of Potsdam, Potsdam, Germany; 4 Center for Cardiovascular Research, Institute of Pharmacology and Toxicology, Charité, Berlin, Germany; 5 Department of Anesthesia, Critical Care and Pain Medicine, Massachusetts General Hospital, Harvard Medical School, Boston, MA, USA; 6 Pathology, Bayer HealthCare, Wuppertal, Germany; 7 Institute of Pharmacy, Martin Luther University, Halle, Germany

## Background

A direct pharmacological stimulation of soluble guanylate cyclase (sGC) is an emerging therapeutic approach to the management of various cardiopulmonary disorders associated with endothelial dysfunction. Novel sGC stimulators, including riociguat (BAY 63-2521), have a dual mode of action: they sensitize sGC to endogenously produced nitric oxide (NO) and also directly stimulate sGC independently of NO. Little is known about their effects on tissue remodelling and degeneration and survival in experimental malignant hypertension.

## Results

Mortality, hemodynamics and biomarkers of tissue remodelling and degeneration were assessed in Dahl salt-sensitive rats maintained on a high salt diet and treated with riociguat (3 or 10 mg/kg daily) for 14 weeks. Riociguat markedly attenuated systemic hypertension, improved systolic heart function and increased survival (from 33% in the placebo group to 85% in the treated animals). Histological examination of the heart and kidneys revealed that riociguat significantly ameliorated fibrotic tissue remodelling and degeneration. Correspondingly, mRNA expression of the pro-fibrotic biomarkers osteopontin (OPN), tissue inhibitor of matrix metalloproteinase-1 (TIMP-1) and plasminogen activator inhibitor-1 (PAI-1) in the myocardium and the renal cortex was attenuated by riociguat. In addition, riociguat reduced plasma and urinary levels of OPN, TIMP-1, and PAI-1.

## Conclusion

Stimulation of sGC by riociguat markedly improves survival and attenuates systemic hypertension and systolic dysfunction, as well as fibrotic tissue remodelling in the myocardium and the renal cortex in a rodent model of pressure and volume overload. These findings suggest a therapeutic potential of sGC stimulators in providing organ protection in diseases associated with impaired cardiovascular and renal functions.

